# Acoustic Emission and K-S Metric Entropy as Methods to Analyze the Influence of Gamma-Aluminum Oxide Nanopowder on the Destruction Process of GFRP Composite Materials

**DOI:** 10.3390/ma16237334

**Published:** 2023-11-25

**Authors:** Katarzyna Panasiuk, Krzysztof Dudzik, Grzegorz Hajdukiewicz, Norbert Abramczyk

**Affiliations:** Faculty of Marine Engineering, Gdynia Maritime University, 81-225 Gdynia, Poland; k.dudzik@wm.umg.edu.pl (K.D.); g.hajdukiewicz@wm.umg.edu.pl (G.H.); n.abramczyk@wm.umg.edu.pl (N.A.)

**Keywords:** acoustic emission, K-S metric entropy, nano-additives, GFRP, tensile test

## Abstract

Composites are materials that are widely used in industry, including yachting, railway and aviation. The properties of these materials can be modified by changing the type of reinforcement, the type of matrix, as well as the use of additives in the form of fillers and nanofillers that improve their mechanical or specific parameters. Due to the fact that these materials are often used for important structures, computational models using FEM tools may not be sufficient to determine the actual strength parameters, and what is more, to check them during operation. When designing structures made of composite materials, it is necessary to use high safety factors due to their behavior under several different types of loads, which is still difficult to determine precisely. This situation makes these structures much heavier and characterized by much higher strength properties than those that would actually be needed. In this article, the Kolmogorov-Sinai (K-S) metric entropy was used to determine the transition from the elastic to the viscoelastic state in GFRP (glass fiber reinforced polymer) composite materials without and with the addition of nanoaluminum, during a static tensile test. Additionally, the acoustic emission method was used during the research. This signal was further processed, and graphs were made of the number of events and the amplitude as a function of time. The obtained values were plotted on tensile graphs. The influence of the nano-filler on these parameters was also analyzed. The presented results show that it is possible to determine additional parameters affecting the strength of the structure for any composite materials.

## 1. Introduction

Due to their properties, composite materials are used as construction materials in many industries, from windmills, wagons or their parts and airplanes to yachts. Due to the easy modification of the strength properties of these materials, scientists are developing newer technologies and compounds to obtain even better properties. One such possibility is to combine laminates with additives in the form of nanofillers to create new materials called nanocomposites. Nanocomposites are materials in which at least one component has dimensions on the nanometric scale, which is called a nanofiller.

Nanofillers can be classified according to their chemical nature and type of physical structure, but most often they are classified according to the shape of the particles [1,2,3]. The attractiveness of nanocomposites results from the fact that the polymer matrix and the nanofiller interact with each other already at the molecular level. Thanks to this, a nanofiller with dimensions below 100 nm, added in a small amount to the matrix (usually a few percent), can significantly change the selected properties of the composite material. Research began with other aluminosilicates and substances with a lamellar structure, using a number of polymers as polymer matrices [4,5,6,7]. Nanocomposites with aluminosilicate fillers are currently used for the production of car engine details, in the aviation and space industries, etc. [8,9,10]. In article [11], composites based on epoxy resin/graphene oxide (GO) and epoxy resin/reduced graphene oxide (rGO) were tested in terms of thermo-mechanical properties, with emphasis on the influence of the chemical groups present on surfaces enriched with nano-additives. Studies have shown that the presence of oxidizing groups on GO contributes to the improvement of exfoliation, intercalation and distribution of GO sheets in composites compared to composites based on rGO. In study [12], metal-based nano-additives were incorporated into the PLA matrix, thus examining their influence on the surface properties of the antibacterial activity and mechanical properties of the PLA nano-additive film. The main aim of the research was to determine how the addition of nanoparticles to PLA during the extrusion process affects the chemical composition, morphology and wettability of the surface and its further impact on the antibacterial effectiveness and mechanical properties of PLA-NP. The article [13] focused on the influence of multi-wall carbon nanotubes on the mechanical and electrical properties of epoxy resins and epoxy composites. By using carbon nanotubes as polymer reinforcement, higher tensile strength values and higher deformation percentages were achieved. Most articles mainly focus on graphene nano-additives [14]. In study [15], the influence of the weight percentage of aluminum oxide nanoparticles (0.25–0.5–0.75–1 wt) and the particle size of 60 nm mixed with a commercial epoxy resin (Kemapoxy 150) was used. Bending, hardness, tensile, erosion, water absorption and TGA (thermogravimetry) tests were performed. The experimental results showed an improvement in mechanical behavior using 0.25 wt% particles for both flexural strength and wear resistance by 7 and 67% compared to pure epoxy. The research aimed to dope two nanometal oxides with high-density polyethylene (HDPE) using roller mixing and thermal pressing techniques. The weight fractions of the nanofillers ranged from 2.0% to 8.0% in the obtained composite. Interlayer shear strength (ILSS) was performed on a combination of nanofiller additives (Al_2_O_3_ and TiO_2_) in the polymer composite. It was observed that the simultaneous presence of Al_2_O_3_ and TiO_2_ nanofillers improves ILSS [16,17,18].

Due to the continuous search for materials that can improve the mechanical properties of composites [19,20,21], in this article it was decided to test composites based on glass fibers (GFRP) with the addition of the gamma-aluminum oxide nanopowder nanofiller. In order to more precisely visualize the results and determine the impact of the nanoadditive on the destruction process of composite materials, tools such as the acoustic emission method and K-S metric entropy were used for analysis [22]. 

The static tensile test of composite materials itself allows for the determination of parameters related to the strength of the materials, but it does not determine the changes that occur in the structure of these materials, such as, among others, in the case of metals, the yield point. The characteristics of the destruction process of composite materials during loading are still not known and adequately characterized. The use of acoustic emission signals during loading makes it possible to observe phenomena occurring in composite materials and, after a broader analysis, to also identify them. It is necessary to use appropriate parameters, such as the amplitude and number of events. Kolmogorov-Sinai metric entropy, based on deformation parameters, allows for determining the place where a change in the nature of deformation occurs (structural change). The combination of these two methods and appropriate correlation allows results to be obtained that can contribute to a completely different approach to the design of composite structures, not only using safety factors, but also real values.

## 2. Materials and Methods

Acoustic emission used simultaneously with a static tensile test, among many methods for detecting damage in composite materials, is very sensitive. It enables the study of deformation in the elastic or plastic range [23,24,25]. It allows for the monitoring and identification of damage, from the microscopic level to macroscopic changes, including the total destruction of the tested material [26,27,28]. The use of mechanical tests and acoustic emission allows for the detection and identification of individual destruction processes of composite materials, such as fiber breakage, matrix-reinforcement interface cracking and the cracking of the matrix. For this purpose, AE signals recorded during tests should be analyzed. The basic parameters describing these signals are amplitude, RMS, number of events, rise time, duration and energy [29,30]. The events that occur while loading the sample are impossible to observe, but they can be heard. Depending on the type of destruction process, these are, of course, different frequencies and different amplitudes. Taking into account, for example, the process of matrix destruction, we are dealing with brittle cracking that does not have large amplitude values. The process of the destruction of the fibers themselves is characterized by a larger number of events related to the cracking of both the fibers and the matrix as well as the much longer duration of these events. This is also related to the modulus of the elasticity of the fibers, which is much larger than the modulus of the elasticity of the matrix. The above conclusions are not based on theory or literature, but own research [31]. For more detailed damage monitoring, it is recommended to include an appropriate tool or signal in the analysis. Several studies conducted by the authors of this article [22,31,32,33] show that the use of the acoustic emission method combined with mechanical tests allows for obtaining much more information about composite materials than in the case of standard methods.

Additionally, in addition to using the acoustic emission method in the research, it was decided to use a statistical method based on Kolmogorov-Sinai (K-S) metric entropy. This method assumes that the qualitative changes occurring at the structural threshold separating the elastic state from the plastic state correspond to a specific measurement point. Energy dissipation occurs in the system, and the deterministic chaos of data associated with this phenomenon causes entropy variability [22]. The preparation of input data and the accuracy of measurement tools are extremely important when using this method [34]. The minimum value of metric entropy near the transition from the elastic to the plastic state is determined by the point separating the individual process states. The value of the stress corresponding to the transition of the tested material from the elastic to the plastic state is determined on the basis of the ‘critical’ point [35,36]. This method, together with its application based on research on composite materials and others, has been previously described in detail [22].

Polyester-glass laminates with the addition of a filler in the form of gamma-aluminum nanopowder were used for the tests. In order to reduce the risk of improper mixing of the resin and the nanofiller, the use of infusion methods and improper infiltration of resin into the materials, e.g., by stopping or a significant change in viscosity, the manual lamination method was used. Polimal 1094-AWTP polyester resin and glass mat with a weight of 450 g/m^2^ were used. The matrix additives, such as recycled filler and nanofiller, were combined by physical mixing using a mixer. Two research materials were made: A0—without the addition of nanofiller, A2—with 2% nanofiller content. Table 1 shows the % content (by weight) of the combined ingredients.

**Figure 1:**
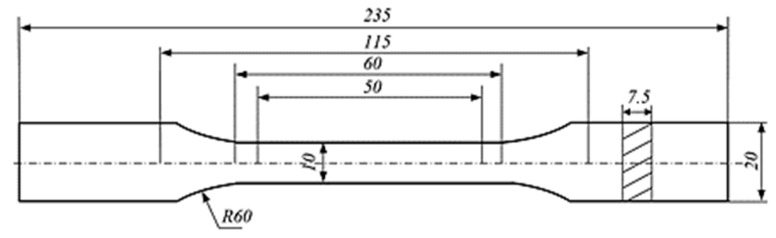
Dimensions of the specimens for the static tensile test in accordance with standard [31,37].

**Figure 2:**
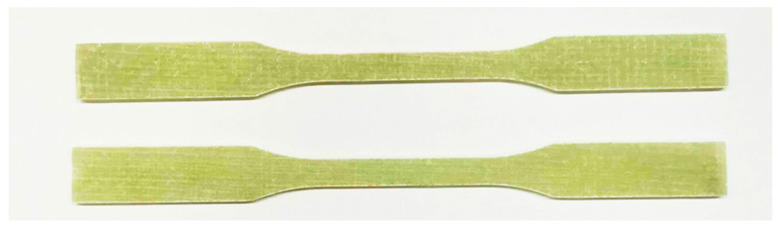
Selected specimens for testing.

The composite materials created in the manufacturing process were prepared for static tensile testing in accordance with the standard [37]. In order to obtain the appropriate shape of the specimens and minimize the influence of cutting temperature, the water-cutting method was used. 30 samples were cut from each material. Figure 1 shows the dimensions of the specimen, in accordance with the standard. Figure 2 shows a photo of the specimens prepared for the test.

**Figure 3:**
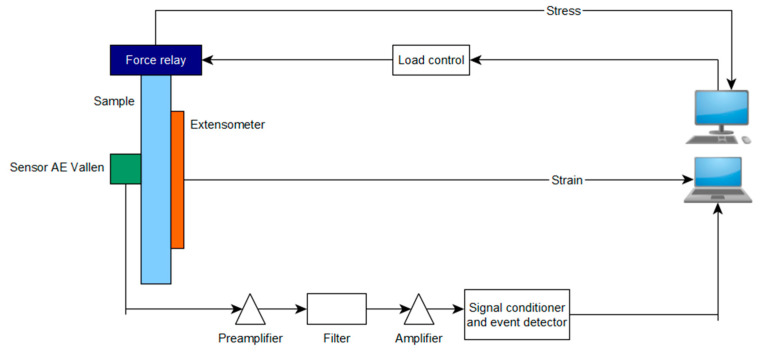
Diagram of measuring station [31].

The tests were carried out using AE measuring instruments (VallenSysteme GmbH, Icking, Munich, Germany) and a universal testing machine from Zwick Roel (Zwick Roell Group, Ulm, Germany). A piezoelectric sensor was installed on the tested specimens, which records the acoustic waves generated inside the material. These waves were converted by the sensor into an electrical signal, and then recorded in digital form by the recorder. The recorded signal was further processed and graphs of the effective value of the electrical signal (RMS), amplitude and hits as a function of time were plotted [22]. The instruments consisted of the AMSY-6 measurement system (VallenSysteme GmbH, Icking, (Munich), Germany); four ASIP-2 measurement (VallenSysteme GmbH, Icking, Munich, Germany) cards for recording and processing the AE signal; and VS-150-M sensors. AE research was performed using a set composed of a single channel recorder, USB AE Node, type 1283 with bandpass 20 kHz–1 MHz; a preamplifier with bandpass 75 kHz–1.1 MHz; AE-Sensor VS 150 M (with a frequency range of 100–450 kHz); and a computer with AE Win for USB Version E5.30 software for recording and analyzing AE data. The software used in the AE data acquisition was VisualAE (VallenSysteme GmbH, Icking, (Munich), Germany). Figure 3 shows a diagram of the measurement system [31].

**Figure 4:**
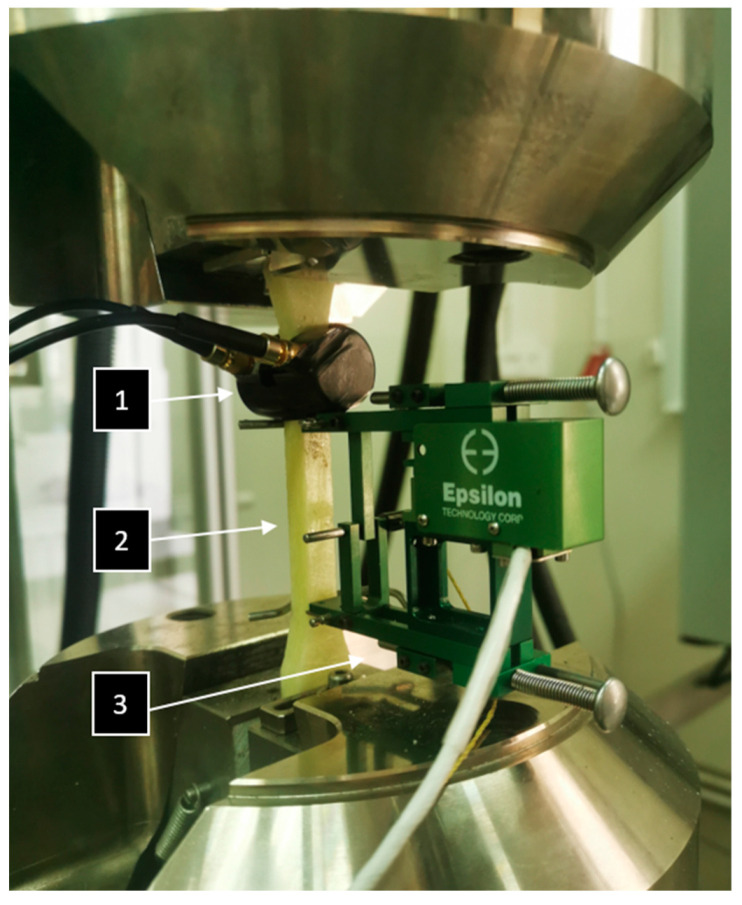
Specimen being tested (2) with an AE sensor (1) and an extensometer (3).

In order to obtain accurate measurements and use the K-S metric entropy method, an extensometer was used to accurately measure the strain. The AE sensor was attached to the specimen using an elastic band, and a coupling fluid was used between the sensor and the specimen surface. Figure 4 shows the specimen with the AE sensor and extensometer.

## 3. Results and Discussion

**Figure 5:**
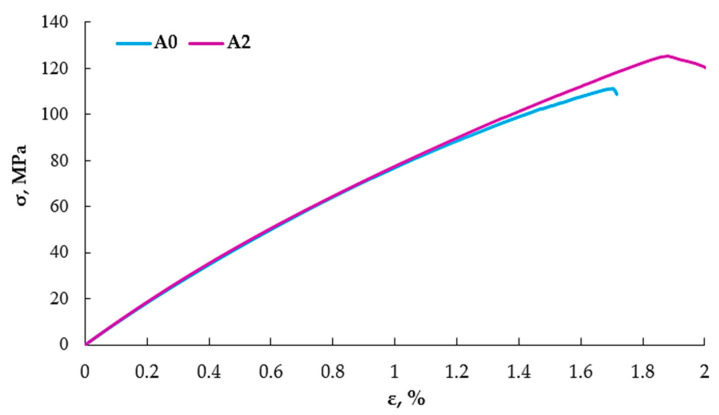
Tensile diagrams of selected specimens.

During the static tensile test, 30 specimens from each material were tested. Table 2 presents a summary of the obtained results (average values) and Figure 5 shows graphs for two selected specimens.

Based on the results obtained from the static tensile test alone, it can be concluded that the nanofiller partially replacing the reinforcement improves the mechanical properties. As a result of the addition of the nanofiller, the tensile strength—σ—increased by about 11%, the elastic modulus—E—by about 3%, and the strain—ε—by about 9%. Despite the lack of chemical modifiers, the aluminum nanofiller combined with the resin did not adversely affect the strength parameters. It is worth mentioning that the addition of aluminum was 2% and it increased the strength properties by 9%. Therefore, it would be worth verifying in subsequent studies how larger amounts of the nanoadditive would affect the strength parameters.

**Figure 6:**
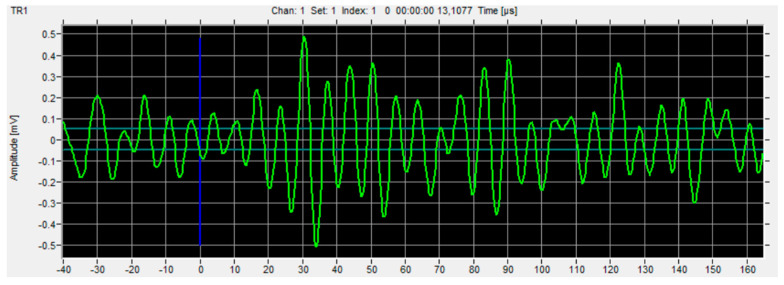
View of the signal amplitude change as a function of time—raw signal, without processing. (Green line is graph of an amplitude, blue line is a zero line).

**Figure 7:**
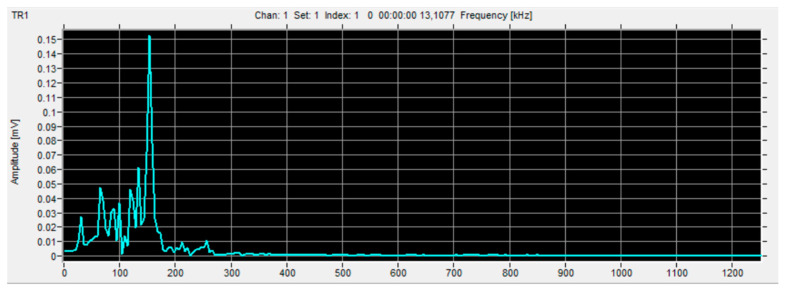
Changes in signal amplitude as a function of frequency—after FFT analysis.

**Figure 8:**
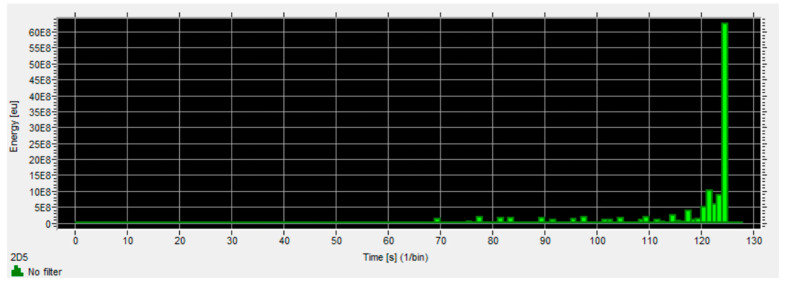
Change of signal energy over time.

Based on previous studies [22,31,32,33], from among the many types of data obtained from acoustic emissions such as hits, RMS, amplitude, duration, etc., it was decided to select the parameter regarding the number of events and the analysis was based on the results of this parameter. Figure 6, Figure 7 and Figure 8 show example charts obtained directly from the VisualAE software (Version 6.2).

FFT analysis is performed to find the characteristic value of the signal frequency for specific destruction mechanisms: matrix cracking, delamination, fiber cracking, etc. Thanks to preliminary research and subsequent comparison of these frequencies, it is possible to clearly determine the phenomenon generating a given signal.

**Figure 9:**
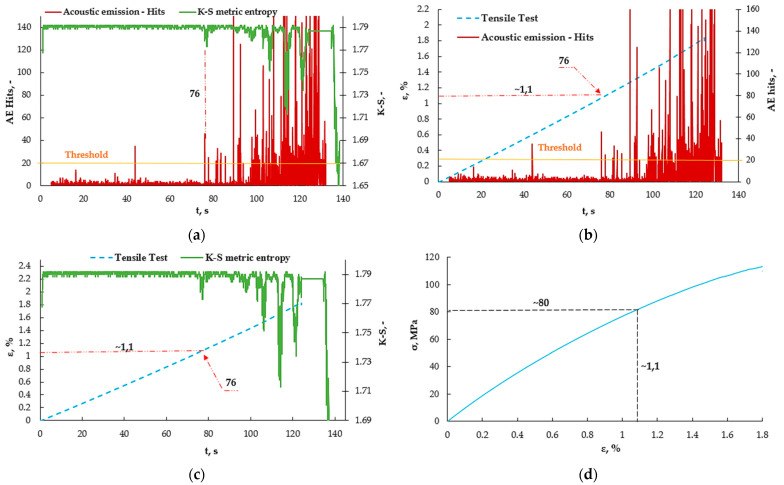
Results obtained from measurements using the acoustic emission method, using the K-S metric entropy for selected sample A0: (**a**) AE hits (t) plot plotted on the K-S (t) entropy graph, (**b**) graph ε (t) plotted on acoustic emission graph—AE hits (t), (**c**) ε (t) graph plotted on K-S (t) entropy graph, (**d**) obtained values plotted on a σ (ε) graph.

The values obtained using the K-S metric entropy were also plotted on the obtained graphs. The parameters of strain, number of events (AE) and metric entropy are plotted as a function of time, since the K-S metric entropy is calculated based on the strain values obtained from the extensometer. In earlier work, the K-S metric entropy itself was presented in the charts as a function of measurement points, but for the purpose of comparing these results, the same reference point had to be adopted. Therefore, processing this data requires making at least three graphs to present the obtained values. Figure 9 shows the results obtained for an example sample without the addition of a nanofiller.

**Figure 10:**
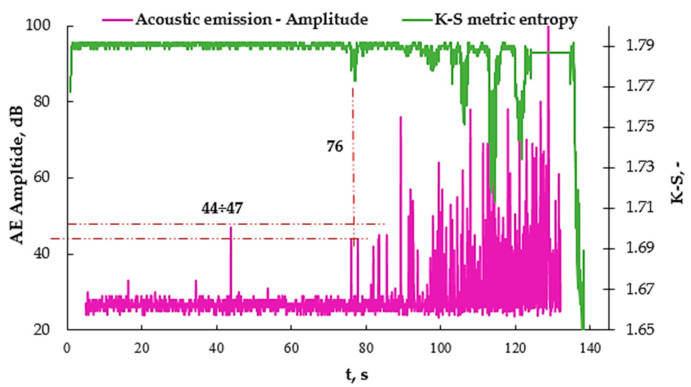
AE amplitude (t) graph plotted on the K-S (t) entropy graph for selected sample A0.

Figure 9a shows the values obtained from the acoustic emission as well as from the K-S metric entropy method. There is a noticeable common point for the decrease in entropy and the increase in the number of events at approximately the 76 s mark of the test. The previous values of the number of AE events were defined as the threshold because their values are comparable from the very beginning of the measurement. Except for one moment at 46 s, an additional graph was made to verify the amplitude value (Figure 10). The measurement using the acoustic emission method was started with a slight delay, hence the shift of the graph by a few seconds. Figure 9b shows the graphs of the number of AE events as a function of time, plotted on the strain ε (t) graphs, which made it possible to read the strain value at which this increase occurs. The strain for 76 s was approximately 1.1%. The same was done in the case of the K-S metric entropy (Figure 9c) and the same strain value was read from the graph. In Figure 9d, the value of the obtained strain for 76 s was plotted on the graph obtained from the static tensile test σ (ε), thanks to which the stress value was determined to be approximately 80 MPa. Earlier use of metric entropy in research, among others, aluminum alloy [36] made it possible to determine the structural change—the transition from the elastic to the plastic state in metals. In the case of composites, it is difficult to clearly define the point at this type of transition. In article [31], the emission results to determine the structural changes in the composite material were based on the amplitude, the values of which correspond to the number of events. Figure 10 shows the AE amplitude graph plotted on the K-S metric entropy graph.

The amplitude value at which the characteristic increase occurs (43 s) is approximately 47 dB. However, it can be omitted, taking into account the results obtained in previous studies and verified with those values that coincide with the K-S metric entropy (44 dB). In article [31], a detailed analysis of the destruction process using GFRP was carried out but with a different weight of the material used (350 g/m^2^), which resulted in a reduction in the tensile strength of this material; however, by analyzing the results presented in Table 3, they can be compared with each other for the purpose of characterizing the point at 76 s, corresponding to approximately 1.1% deformation.

**Figure 11:**
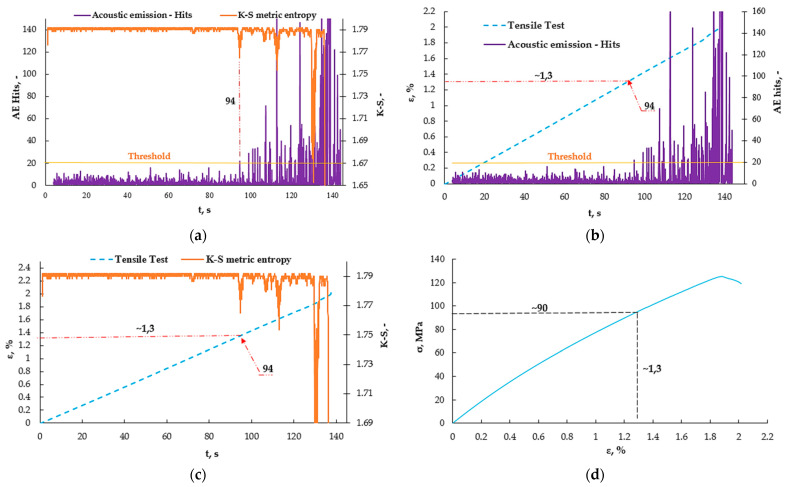
Results obtained from measurements using the acoustic emission method, using the K-S metric entropy for selected sample A2: (**a**) AE Hits (t) graph plotted on the K-S (t) entropy graph, (**b**) graph ε (t) plotted on acoustic emission graph—AE Hits (t), (**c**) ε (t) graph plotted on K-S (t) entropy graph, (**d**) obtained values plotted on a σ (ε) graph.

Analyzing the results obtained for the amplitude at 76 s of the test, corresponding to 44 ÷ 47 dB, and the values in Table 3, it can be concluded that these two points indicate the beginning of the cracking of the matrix, with simultaneous fiber delamination. Hence, it can be characterized as the initiation of permanent and irreversible changes in the composite material. Figure 11 shows a similar analysis but using an aluminum nanoadditive.

**Figure 12:**
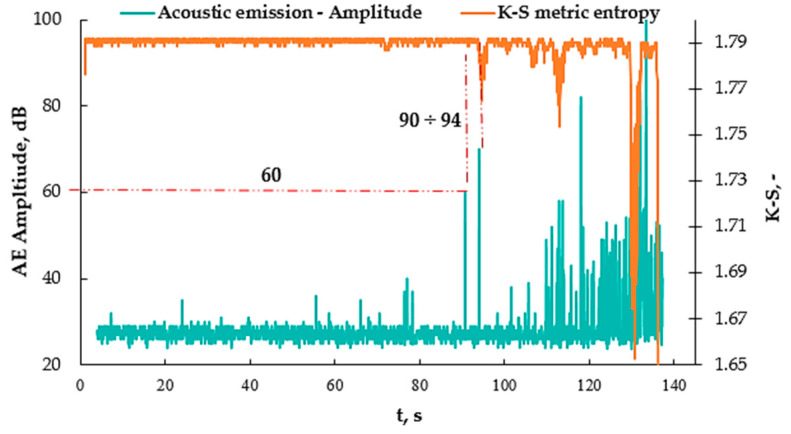
AE Amplitude (t) graph plotted on the K-S (t) entropy graph for selected sample A2.

Figure 11a shows the values obtained from the acoustic emission as well as from the K-S metric entropy method. There is a noticeable common point for the decrease in entropy and the increase in the number of events at approximately the 94 s mark of the test. Figure 11b shows graphs of the number of AE events as a function of time, plotted on strain-rate—ε (t) graphs, which made it possible to read the strain value at which this increase occurs. The strain for 86 s was approximately 1.3%. The same was done for the K-S metric entropy (Figure 11c) and a strain value of approximately 1.3% was read from the graph. In Figure 11d, the value of the obtained strain for 86 s was plotted on the graph obtained from the static tensile test σ (ε), thanks to which the stress value was determined to be approximately 90 MPa. Figure 12 shows the AE amplitude graph plotted on the K-S metric entropy graph.

Analyzing the graph (Figure 12) and the values in Table 3, it can be concluded that the addition of nanoaluminum to the resin significantly influenced the elasticity of the composite, extending the range of deformations for the matrix itself. It was increased from 80 MPa to 90 MPa, or by approximately 13%, by adding just 2% of the nanoadditive to the resin.

## 4. Conclusions

Nanoadditives are materials that are used to increase the strength parameters of composite materials. They may influence the variability of a number of parameters, such as tensile strength or strain. In this article, a 2% aluminum nanoadditive was used, thus replacing the reinforcement, in order to determine its impact on the strength parameters of the composite comparing the modified material to material without the additive. Using the K-S metric entropy method and the acoustic emission method, its influence on the mechanical and strength parameters was verified, not only on the basis of a static tensile test. The nanofiller, which partially replaces the reinforcement, improves the mechanical properties. As a result of the addition of the nanofiller, the tensile strength—σ—increased by about 11%, the elastic modulus—E—by about 3%, and the strain—ε—by about 9%. Despite the lack of the addition of chemical modifiers, the nanofiller had a positive effect on the mechanical properties. Using the K-S metric entropy method and the acoustic emission method, the values at which permanent deformations occur in the composite were determined, including the cracking of the matrix or delamination of the fibers. For the composite without the addition of aluminum, a value of 80 MPa was obtained, while thanks to the nano-addition of aluminum—an increase of 13% and a value of 90 MPa were obtained. Application of the abovementioned methods, as additional tests for new materials, allows for the determination of many more values that are particularly important when designing structures. Static tensile testing of composite materials, in most cases, provides a tensile strength value but does not involve yield stresses, commonly used to characterize metals. The composite material and its behavior during loading are not as known and characterized. The analysis of AE signals recorded during loading enables not only the observation of phenomena that occur during the material degradation processes, but also their identification. In laboratory tests, appropriate sensitivity thresholds should be determined for characteristic signal parameters, e.g., the amplitude and number of events. Knowing the values for materials that cause permanent changes allows for the determination of the permissible stress values for composites already during the design stage. This opens a new perspective on the research of composite materials and confirms the belief that there are research methods that allow for the verification of structural changes in composite materials, not only using tools at the micro-scale, such as SEM, but also at the macro scale.

## Figures and Tables

**Table 1:** List of A0 and A2 composite ingredients.

Sample	Resin	Matrix	Nanoadditive
	%	%	%
**A0**	60	40	0
**A2**	60	38	2

**Table 2:** Summary of the results obtained from the static tensile test (30 specimens).

Sample	σ	E	ε
	MPa	MPa	%
A0	114.13	8927	1.71
Standard deviation	5.32	521	0.10
A2	128.26	9156	1.88
Standard deviation	8.40	434	0.12

**Table 3:** Summary of the analysis of the degradation of the materials [31].

Signal Type	Amplitude	Stress
	dB	MPa
Matrix deformation	31–41	10–40
Cracking of matrix, delamination of fibers	42–50	38–50
Cracking of fibers	>50	51–110

## Data Availability

Data available on request due to restrictions eg. privacy or ethical. The data presented in this study are available on request from the corresponding author. The data are not publicly available due to its huge amount.
